# Microstructure and Nonohmic Properties of SnO_2_-Ta_2_O_5_-ZnO System Doped with ZrO_2_

**DOI:** 10.1155/2014/754890

**Published:** 2014-01-20

**Authors:** Xiuli Fu, Feng Jiang, Ruichao Gao, Zhijian Peng

**Affiliations:** ^1^School of Science, Beijing University of Posts and Telecommunications, Beijing 100876, China; ^2^School of Engineering and Technology, China University of Geosciences, Beijing 100083, China

## Abstract

The microstructure and nonohmic properties of SnO_2_-Ta_2_O_5_-ZnO varistor system doped with different amounts of ZrO_2_ (0–2.0 mol%) were investigated. The proposed samples were sintered at 1400°C for 2 h with conventional ceramic processing method. By X-ray diffraction, SnO_2_ cassiterite phase was found in all the samples, and no extra phases were identified in the detection limit. The doping of ZrO_2_ would degrade the densification of the varistor ceramics but inhibit the growth of SnO_2_ grains. In the designed range, varistors with 1.0 mol% ZrO_2_ presented the maximum nonlinear exponent of 15.9 and lowest leakage current of 110 **μ**A/cm^2^, but the varistor voltage increased monotonously with the doping amount of ZrO_2_.

## 1. Introduction

SnO_2_ varistors are semiconducting ceramic devices, which possess nonlinear voltage-current characteristics due to their grain boundary effects formed commonly by sintering SnO_2_ powder with minor additives (impurity). Due to their excellent energy handling capabilities, they can be applied extensively to protect electronic circuits, various semiconductor devices, and electric power systems from dangerous abnormal transient overload [1, 2].

The first impurity-doped SnO_2_ varistor was reported by Glot and Zlobin [3], and Pianaro et al. also made great contributions to the knowledge of varistor behavior of impurity-doped SnO_2_ ceramics [4]. Through a series of studies on SnO_2_-based varistors for decades, it is well known that an excellent SnO_2_ varistor system consists of three kinds of dopants: resistance reducers (varistor forming oxide, VFO), densifiers, and modifiers, respectively [5]. To date, the commonly applied VFOs are Nb^5+^ [6–8] or Ta^5+^ [9–11], which possesses high chemical valence and is soluble in SnO_2_ grains to decrease the grain resistivity; the densifier is insoluble ion of low chemical valence that will segregate at SnO_2_ grain boundary regions to promote the densification by producing oxygen vacancies, for example, Co^2+^ [4, 6, 7, 9, 11], Mn^2+^ [12, 13], and Zn^2+^ [8, 14], and the modifiers can effectively improve the electrical properties of the varistors, such as Cr^3+^, Fe^3+^, Cu^2+^, and rare earth elements [6–9, 15, 16].

Moreover, during modern ceramics processing, high energy attrition milling and ZrO_2_ grinding media were often applied. As a result, Zr^4+^ contamination in ceramic samples is a common phenomenon. However, up to now, no literature about the role of Zr^4+^ ion (ZrO_2_) in SnO_2_-based varistors has been reported.

Recently, we optimized a SnO_2_-Ta_2_O_5_-ZnO varistor system, which presents varistors of good nonlinear properties but very low varistor voltage [17]. Based on it, in the present study, SnO_2_-Ta_2_O_5_-ZnO-based varistor system was doped with ZrO_2_ (0–2.0 mol%), and the effect of ZrO_2_ doping on the microstructure and nonohmic properties of SnO_2_-Ta_2_O_5_ based varistors was investigated. To our surprise, varistors with fully dense structure and high breakdown voltage could be obtained.

## 2. Experimental Procedure

### 2.1. Sample Preparation

The samples were prepared using a conventional ceramic processing method with a nominal composition of (99.45-*x*) mol% SnO_2_ + 0.05 mol% Ta_2_O_5_ + 0.5 mol% ZnO + *x* mol% ZrO_2_ (*x* = 0, 0.25, 0.5, 1.0, 2.0). All the oxides were raw powders of analytical grade. At beginning, the raw powders were mixed in deionized water and ball-milled in polyethylene bottle for 24 h with 0.5 wt% of PVA as binder and highly wear-resistant ZrO_2_ balls as grinding media. Subsequently, the obtained slurries were dried at 110°C in an open oven. After drying, the powder chunks were crushed into fine powders, sieved, and pressed into pellets of 6 mm in diameter and 1.5 mm in thickness under a pressure of 40 MPa. Then, the pressed pellets were sintered at 1400°C for 2 h in a Muffle oven by heating at a rate of 300°C/h and cooling naturally. To measure the electrical properties, silver electrodes were prepared on both surfaces of the sintered disks by heat treatment at 500°C for half an hour.

### 2.2. Materials Characterization

The density of the samples was measured by Archimedes method according to international standard (ISO18754). Their crystalline phases were identified by X-ray diffractometer (XRD, D/max2550HB+/PC, Cu K*α*, and *λ* = 1.5418 Å) through a continuous scan mode with speed of 8°/min. The microstructure was examined on the fresh fracture surfaces of the samples via a scanning electron microscope (SEM, Tescan XM5136). And the average size of SnO_2_ grains in the samples was determined using linear intercept method from the SEM images.

A high-voltage source measurement unit (Model: CJ1001) was used to record the characteristics of the applied electrical field versus current density (*E*-*J*) of the samples. The varistor voltage (*V*_*B*_) was determined at 1 mA/cm^2^ and the leakage current (*I*_*L*_) was the current density at 0.75*V*_*B*_. Then, the nonlinear coefficient (*α*) was obtained by the following equation:

(1) $$\begin{array}{c} \alpha =\frac{\log\left(J_{2}/J_{1}\right)}{\log\left(E_{2}/E_{1}\right)}=\frac{1}{\log\left(E_{2}/E_{1}\right)}, \end{array}$$

where *E*_1_ and *E*_2_ are the electric fields corresponding to *J*_1_ = 1 mA/cm^2^ and *J*_2_ = 10 mA/cm^2^, respectively.

## 3. Results and Discussion

### 3.1. Composition and Microstructure

**Figure 1:**
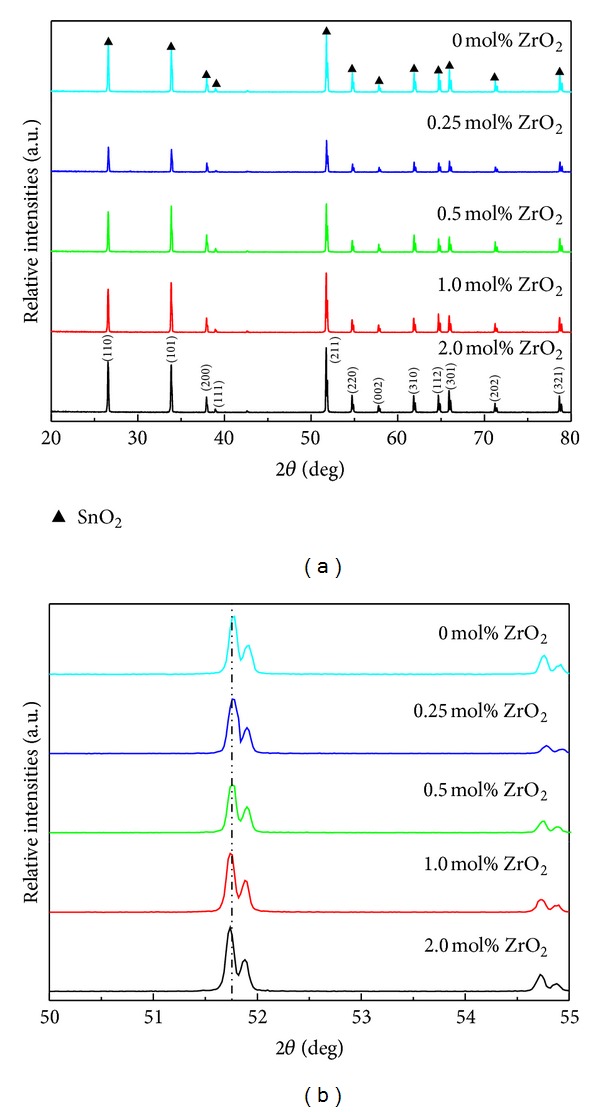
XRD patterns of the as-prepared SnO_2_-Ta_2_O_5_-ZnO-based varistor ceramics doped with different amounts of ZrO_2_: (a) five of the samples and (b) magnified view in 2*θ* region of 50–55°.

Figure 1 illustrates the XRD patterns of the as-prepared SnO_2_-Ta_2_O_5_-ZnO-based varistor ceramics doped with different amounts of ZrO_2_. All the sharp diffraction peaks were assigned, corresponding to the (110), (101), (200), (111), (211), (220), (002), (310), (112), (301), (202), and (321) reflections of SnO_2_ cassiterite phase (JCPDS card no. 77-0451). No extra phases were identified, possibly because the doping levels of the additives were too low to be detected in XRD limits. And, because of the same ionic valence and almost no radius difference between Sn^4+^ (0.071 nm) and Zr^4+^ (0.072 nm) ions, the doped ZrO_2_ is fully soluble in SnO_2_ lattice, which can be seen from almost the same positions of XRD diffraction peaks of the prepared samples as shown in Figure 1(b) in a close view to the patterns in 2*θ* from 50 to 55°. As for the splitting of the XRD peaks in the figure, it might be due to the peak doublet of K-alpha 1 and K-alpha 2.

**Figure 2:**
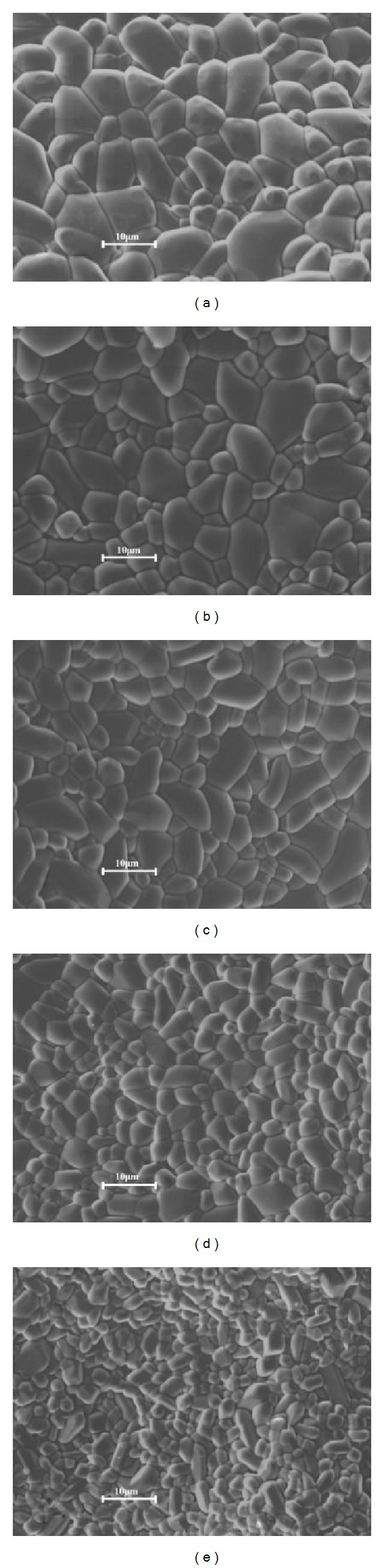
Typical SEM images on fracture surfaces of the as-prepared SnO_2_-Ta_2_O_5_-ZnO-based varistor ceramics doped with different amounts of ZrO_2_: (a) undoped, (b) 0.25, (c) 0.5, (d) 1.0, and (e) 2.0 mol%.

SEM images of the as-prepared SnO_2_-Ta_2_O_5_-ZnO based varistor ceramics also confirmed the solubility of ZrO_2_ into SnO_2_ lattice (please see Figure 2). The images reveal that, although doped with different amounts of ZrO_2_, the typical microstructure of the samples almost has no change: almost fully dense structure of SnO_2_ grains without any obvious second phases. The detailed microstructural parameters are also summarized in Table 1. With increasing doping amount of ZrO_2_, the density of samples decreases in a very narrow range from 6.93 to 6.80 g/cm^3^ partly because the density of ZrO_2_ (5.89 g/cm^3^) is lower than that of the matrix SnO_2_ (6.95 g/cm^3^), but the relative density of the samples also decreases although also in a very narrow range from 99.8% to 98.2%, which indicates a decreased sample densification and could be attributed to the lower diffusion ability of solid ZrO_2_ particles in SnO_2_ matrix at the designed sintering temperature because the melting point of ZrO_2_ (2680°C) is much higher than that of SnO_2_ (1630°C). Moreover, from these SEM images, it can be clearly seen that, with increasing ZrO_2_ contents in the ceramics, the average size of SnO_2_ grains decreases, which might be owing to the inhibited transportation of SnO_2_ during sintering by the doped ZrO_2_ with lower diffusion ability.

### 3.2. Electrical Properties

**Figure 3:**
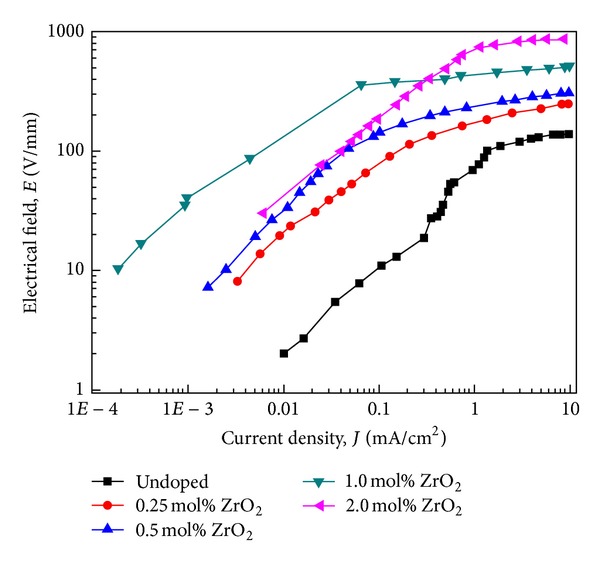
*E*-*J* characteristic curves on a log scale at room temperature of the as-prepared SnO_2_-Ta_2_O_5_-ZnO-based varistors doped with different contents of ZrO_2_.

The *E*-*J* characteristics of the as-prepared SnO_2_-Ta_2_O_5_-ZnO-based ceramic varistors doped with different contents of ZrO_2_ are illustrated in Figure 3, and their corresponding detailed electrical parameters calculated from the *E*-*J* curves are listed in Table 1.

The results indicate that, with increasing doping content of ZrO_2_ up to 1.0 mol%, the nonlinear coefficient of the samples increased up to 15.9, possibly owing to the increased carrier concentration in the varistors, decreased electrical resistivity of SnO_2_ grains and thus enhanced barrier height by doping, and higher number of voltage barriers due to the decrease in grain size, but it would drop down with more ZrO_2_ doped, due to the corresponding less dense sample structure, degraded effective grain boundary, destroyed depletion layer structure, and thus decreased barrier height. The leakage current of the samples presented an opposite trend to that of nonlinear coefficient with ZrO_2_ doping, and the varistors with 1 mol% ZrO_2_ presented the lowest leakage current, 110 *μ*A/cm^2^, which is completely consistent with classic theory on their relationship [18]. Thus, it can be concluded that the optimum doping amount of ZrO_2_ in the proposed SnO_2_-Ta_2_O_5_-ZnO-based ceramic varistor system was 1 mol%. The varistor voltage of the samples increased monotonously with the doping amount of ZrO_2_, which could be mainly attributed to the decreased SnO_2_ grain size, thus increasing the number of grain boundary in unit thickness after doping.

## 4. Conclusions

SnO_2_-Ta_2_O_5_-ZnO varistors doped with different amounts of ZrO_2_ (0–2.0 mol%) were prepared by sintering at 1400°C for 2 h with conventional ceramic processing method. The doping of ZrO_2_ would degrade the densification of the varistor ceramics, but inhibit the growth of SnO_2_ grains. In the designed range, varistors with 1.0 mol% ZrO_2_ presented the maximum nonlinear exponent of 15.9 and lowest leakage current of 110 *μ*A/cm^2^; but the varistor voltage increased monotonously with the doping amount of ZrO_2_.

## Figures and Tables

**Table 1:** Basic physical parameters of SnO_2_-Ta_2_O_5_-ZnO varistor ceramics doped with different contents of ZrO_2_.

Doping amount of ZrO_2_ (mol)	Apparent density (g/cm^3^)	Relative density (%)	SnO_2_ grain size (*μ*m)	*α*	*V*_*B*_ (V/mm)	*I*_*L*_ (*μ*A/cm^2^)
0	6.93	99.8	7.87	4.6	81	660
0.25	6.89	99.2	6.67	6.0	103	560
0.5	6.88	99.1	5.45	8.2	250	220
1.0	6.84	98.6	4.55	15.9	500	110
2.0	6.80	98.2	3.03	11.6	720	170
